# Structural gray matter features and behavioral preliterate skills predict future literacy – A machine learning approach

**DOI:** 10.3389/fnins.2022.920150

**Published:** 2022-09-29

**Authors:** Moana Beyer, Johanna Liebig, Teresa Sylvester, Mario Braun, Hauke R. Heekeren, Eva Froehlich, Arthur M. Jacobs, Johannes C. Ziegler

**Affiliations:** ^1^Department of Education and Psychology, Freie Universität Berlin, Berlin, Germany; ^2^Center for Cognitive Neuroscience Berlin, Freie Universität Berlin, Berlin, Germany; ^3^Centre for Cognitive Neuroscience, Universität Salzburg, Salzburg, Austria; ^4^Department of Biological Psychology and Cognitive Neuroscience, Freie Universität Berlin, Berlin, Germany; ^5^Department of Decision Neuroscience and Nutrition, German Institute of Human Nutrition Potsdam-Rehbrücke, Nuthetal, Germany; ^6^Laboratoire de Psychologie Cognitive, Aix-Marseille Université and Centre National de la Recherche Scientifique, Marseille, France

**Keywords:** MRI, machine learning, reading acquisition, precursors, prediction, children

## Abstract

When children learn to read, their neural system undergoes major changes to become responsive to print. There seem to be nuanced interindividual differences in the neurostructural anatomy of regions that later become integral parts of the reading network. These differences might affect literacy acquisition and, in some cases, might result in developmental disorders like dyslexia. Consequently, the main objective of this longitudinal study was to investigate those interindividual differences in gray matter morphology that might facilitate or hamper future reading acquisition. We used a machine learning approach to examine to what extent gray matter macrostructural features and cognitive-linguistic skills measured before formal literacy teaching could predict literacy 2 years later. Forty-two native German-speaking children underwent T1-weighted magnetic resonance imaging and psychometric testing at the end of kindergarten. They were tested again 2 years later to assess their literacy skills. A leave-one-out cross-validated machine-learning regression approach was applied to identify the best predictors of future literacy based on cognitive-linguistic preliterate behavioral skills and cortical measures in *a priori* selected areas of the future reading network. With surprisingly high accuracy, future literacy was predicted, predominantly based on gray matter volume in the left occipito-temporal cortex and local gyrification in the left insular, inferior frontal, and supramarginal gyri. Furthermore, phonological awareness significantly predicted future literacy. In sum, the results indicate that the brain morphology of the large-scale reading network at a preliterate age can predict how well children learn to read.

## Introduction

Literacy is a multidimensional concept that comprises the ability to read (derive meaning from written symbols) and write (encode meaning through written symbols). Literacy is a key competence in today’s information-driven society. Not surprisingly, difficulties in reading can have far-reaching consequences ranging from low academic achievement (Bruck, 1987; Fletcher and Vaughn, 2009) to emotional problems or even psychiatric disorders (Schulte-Körne, 2010; Livingston et al., 2018). To better understand why some children fail to acquire literacy skills successfully, there has been quite some effort in identifying factors that potentially facilitate or hamper reading acquisition (Hoeft et al., 2007; Perry et al., 2019). This has led to the identification of several precursors of literacy that predict future reading and spelling at the end of kindergarten.

To date, the most robust predictors of literacy prior to formal instruction at school are cognitive-linguistic preliterate skills. The two most reliable skills are rapid automatized naming (RAN) and phonological awareness (PA). RAN tasks assess a child’s speed and accuracy in naming familiar stimuli such as digits, letters, and colors. RAN and fluent reading share many subprocesses, such as saccadic eye movements, lexical access, and the mapping of visual objects onto language representations (Norton and Wolf, 2012). PA refers to the ability to represent, recognize, access, and manipulate any phonological unit within a word. Thus, PA is essential to map orthography onto phonology and hence bootstrap reading acquisition (Ziegler et al., 2014, 2020). The strong link between these two variables and reading acquisition has been repeatedly shown in large-scale cross-linguistic studies both at the concurrent (Ziegler et al., 2010; Landerl et al., 2013) and the longitudinal level (Caravolas et al., 2012; Landerl et al., 2019, 2022). In recent years, there has also been an increasing effort to study neurofunctional (Lohvansuu et al., 2018; Liebig et al., 2020, 2021) predictors of future reading proficiency before the onset of literacy (see Chyl et al., 2021 for a recent review). The overlap of the functional and anatomical neural architecture of reading suggests a close link between brain morphology and function.

Consequently, multiple studies have examined reading-related macrostructural features of the cortex (Linkersdörfer et al., 2012; Richlan et al., 2013; Eckert et al., 2016). However, the results of different studies are far from converging into a uniform picture (Ramus et al., 2018; Chyl et al., 2021). Thus, in the present study, we aimed to compare and weigh the effects of several gray matter macrostructural brain maturation features and behavioral measures of cognitive-linguistic preliterate skills, which were gathered at the end of kindergarten to predict individual literacy skills 2 years later.

A large-scale reading network has been identified in skilled adult readers that can roughly be characterized by two posterior and one anterior stream. The ventral stream is linked to the occipito-temporal cortex and hosts the visual word form area. It is associated with direct orthographic reading strategies (Cohen and Dehaene, 2004; Dehaene and Cohen, 2011). The dorsal stream, located in the temporo-parietal cortex, is primarily devoted to auditory-phonological recoding (Pugh et al., 2000). Both streams converge in the frontal stream linked to the inferior frontal gyrus and insular cortex, among others (Binder et al., 2009; Price, 2012; Martin et al., 2015; Froehlich et al., 2018). However, in the last decades, the modularized view of reading has been increasingly challenged and replaced by a unified view of reading (Price and Devlin, 2011; Braun et al., 2019). According to this view, reading is orchestrated by the large-scale network in a highly distributed and interactive way (Hofmann and Jacobs, 2014; Ziegler et al., 2018). This partly pre-existing network already devoted to language and sensory-motor processing must be fundamentally reorganized during reading acquisition to become responsive to print (Dehaene et al., 2015; Liebig et al., 2017; Dehaene-Lambertz et al., 2018).

Until today, most work examining reading-related brain morphology has focused on altered gray matter volume, hereafter referred to as cortical volume, in impaired compared to neurotypical readers (Ramus et al., 2018). The underlying reason might be the cortical morphology’s potential to be a promising early biomarker of future literacy as it is primarily determined by neurodevelopmental processes *in utero* and is partly confined by genetic heritability (Gilmore et al., 2018). Yet, brain structure undergoes continuing changes that are highly intertwined with changes in cognitive abilities, resulting in interindividual variability (Raznahan et al., 2011; Frangou et al., 2022) and might thus be very well suited to identify subtle differences in the cortical morphology that will affect future for reading acquisition.

However, the results of three coordinate-based meta-analyses showed little consistency across studies (Linkersdörfer et al., 2012; Richlan et al., 2013; Eckert et al., 2016). Nonetheless, they point to decreased cortical volume in all three reading streams. More specifically, bilateral temporo-parietal, left ventral occipito-temporal, left frontal, and bilateral cerebellar regions show volumetric differences in developmental dyslexia. A handful of studies focused on the cortical folding pattern in relation to reading acquisition and dyslexia. Impaired readers seem to exhibit abnormal gyrification in the left occipito-temporal and temporo-parietal cortices (Im et al., 2016; Williams et al., 2018), i.e., the ventral and the dorsal stream. Focusing on single structural features generally bears the risk of overseeing interactions between different anatomical measures. Therefore, Płoński et al. (2017) tested several macrostructural features using a cross-validated (CV) classification algorithm in a large cross-linguistic sample of 8- to 13-year-old children and adolescents. Children with dyslexia displayed increased folding and curvature in left temporo-parietal regions, lower surface area in the prefrontal cortex, i.e., in all three reading streams. This comprehensive analysis revealed the benefit of machine learning approaches and the combination of neuroanatomical measures to identify cortical differences more closely and with greater specificity.

In summary, children and adults with impaired reading show a pattern of decreased cortical volume and surface area paired with abnormal gyrification in diverse regions of the large-scale reading network. The neuroanatomical differences seemed to precede the onset of reading instruction at school. Prereaders later diagnosed with dyslexia showed reduced surface area in all three future reading streams, i.e., the bilateral fusiform gyri (Beelen et al., 2019), the left supramarginal gyrus, and the left inferior frontal gyrus (Hosseini et al., 2013). In contrast, cortical thickness did not differ between groups (Hosseini et al., 2013; Beelen et al., 2019). However, neuroanatomical differences in all three reading streams have not been comprehensively investigated yet. In a longitudinal study examining native German-speaking children, Kuhl et al. (2020) reported mixed results concerning the relationship between macrostructural features and reading. Only abnormal gyrification in the left auditory cortex dissociated preliterate children who developed dyslexia from their typically developing peers. Although a uniform picture is still missing, these studies provide a crucial foundation for characterizing the neural basis of reading difficulties.

All of the above-described studies compared typical and impaired reading acquisition. However, group contrasts can be problematic because there is no consistent definition of impaired reading or dyslexia, and thus different methods and thresholds are utilized to classify children across studies (Francis et al., 2005; Fletcher, 2009). Furthermore, reading performance is a continuous variable and splitting the sample into two categorical groups loses valuable information (Button et al., 2013). A different approach is to directly target the relationship between anatomy and reading in typically developing children using continuous sampling and spanning the entire range of reading proficiency. However, studies investigating macrostructural features of typical reading acquisition are scarce and yield mixed results. Longitudinal studies in emergent to intermediate readers show that decreases in cortical volume in different regions linked to the dorsal and frontal stream (e.g., left inferior parietal cortex, superior temporal gyrus, and precentral gyrus) correlate positively with literacy skills (Houston et al., 2014; Linkersdörfer et al., 2014; Jednoróg et al., 2015). These results indicate that an age-appropriate maturation of the large-scale reading network facilitates reading acquisition from early on. In contrast, Torre and Eden (2019) did not find any relationships between cortical volume and word reading in pre-defined regions of the reading network, neither in a large sample of 404 typical readers (6- to 22-year-old) nor in a subsample of 6- to 9-year-old children.

Similarly, Perdue et al. (2020) reported mixed results. They identified a positive relationship between cortical thickness in the left superior temporal gyrus and word and pseudoword reading in typically developing children (4- to 9-year-old) but did not find a relationship between reading skills and surface area in their whole-brain based analysis. These results were supported by a study that tested Chinese-speaking children and showed that word reading was positively correlated with cortical thickness in bilateral superior temporal gyri, the left inferior temporal gyrus, and the left supramarginal gyrus (Xia et al., 2018).

In all of these studies, neuroanatomical features were correlated with literacy-related skills. Correlational frameworks, however, do not allow for generalization to unseen individuals (Dubois and Adolphs, 2016). Furthermore, correlational approaches with a small sample size are prone to over-fitting both signal and noise (Vul et al., 2009; Dubois and Adolphs, 2016; Elliott et al., 2020; Sui et al., 2020). This limitation can be tackled by using CV methods, in which a training sample predicts performance in an independent data set. Until today, there are only a few landmark studies using CV methods to predict literacy-related skills in continuous samples. For example, Skeide et al. (2016) used a whole-brain kernel ridge regression to test individual differences in cortical volume in several reading-related regions to predict reading speed in 5- to 12-year-old native German-speaking children. Bilateral middle frontal gyri, the left superior temporal gyrus, and the left occipito-temporal cortex were positively associated with reading skills. In addition, clusters in the visual word form area and the left visual cortex were negatively associated with reading speed (Skeide et al., 2016). Thus, crucial regions of the dorsal and ventral stream predicted reading with high precision. Choosing a similar approach (Cui et al., 2018), used large datasets of the Human Connectome Project to predict individual reading comprehension and decoding skills in young adults. More specifically, they performed an elastic net penalized linear regression to predict individual literacy scores based on whole-brain cortical volume. The most critical predictive clusters were located in frontal and subcortical regions. The generalizability of the prediction model was then tested in an independent sample of Chinese children (8- to 13-years-old) with mixed results.

One could summarize the mixed results of the relationship between the macrostructural features and typical reading development in the following way: Firstly, all studies that found a significant relationship between macrostructural brain measures and reading report positive relationships. Secondly, cortical volume, particularly in the temporo-parietal areas (Houston et al., 2014; Linkersdörfer et al., 2014), i.e., in the ventral and dorsal stream, is not only robustly correlated with but also predicts reading performance in children (but see, Torre and Eden, 2019, for an exception). Cortical thickness in all three reading streams, i.e., superior temporal gyrus, supramarginal gyrus, and inferior frontal gyrus, showed a positive relationship in typical reading children (Xia et al., 2018; Perdue et al., 2020). Until today, there is only one study testing the effect of the surface area on typical reading acquisition, which did not find any significant correlation (Perdue et al., 2020). However, replication is still outstanding. Thus, the goal of the present study was to add new evidence to the still preliminary data on the relationship between macrostructural features and reading acquisition. More specifically, we aimed at predicting future literacy in a sample of German-speaking kindergarten children using continuous sampling and CV prediction modeling. Several aspects distinguish the present research from the two studies summarized above. Firstly, we analyzed longitudinal data to predict the literacy skills of kindergarten children 2 years later instead of examining concurrent brain-behavior relationships (see Ramus et al., 2018, for promises and pitfalls). Secondly, we included several macrostructural features and compared their relative importance. Thirdly, we used both structural and behavioral information (RAN and PA tested at the end of kindergarten) in the CV models to investigate whether or not, and if so, to what extent adding neuroanatomic data would improve the prediction of future literacy over and above cognitive-linguistic preliterate skills. For that, we obtained cortical volume, surface area, and local gyrification (lGI) from structural scans of preliterate children at the end of kindergarten. The reasoning behind our choice was the following: Firstly, we included cortical volume as one of the most widely tested cortical measures to show the validity of our data and see if we could replicate the well-established pattern using a CV-algorithm. Secondly, we aimed to re-test surface area as this feature was so far tested only once in a continuous sample. Thirdly, we added the lGI that was previously only tested in group comparisons (Williams et al., 2018), where it yielded new and promising insights. Thus, we decided to incorporate this relatively new measure into the present analysis.

The cohort of children was then tested again at the end of the second year of primary school to assess their literacy skills. An elastic net regularized regression was applied to predict future literacy ability. The model was based on cognitive-linguistic preliterate skills and anatomical markers of the cortical surface in pre-defined regions of the “future” reading network. To the best of our knowledge, this is the first study to apply a continuous machine-learning approach to predict future literacy abilities.

Based on previous pediatric neuroimaging, we hypothesized that cortical volume, surface area, and lGI in all three (future) reading streams gathered at a preliterate age would predict literacy 2 years later. More specifically, we expected a crucial contribution of the cortical volume of the occipito-temporal (Skeide et al., 2016) and temporo-parietal cortices (Houston et al., 2014; Linkersdörfer et al., 2014; Jednoróg et al., 2015) to the prediction of future reading skills. Our hypothesis regarding the lGI was less specific, as this feature has not yet been tested with a continuous approach. However, referring to the promising results in group-based approaches (Williams et al., 2018), we expected that the gyrification pattern in the occipito-temporal cortex might predict future literacy. Similarly, reduced surface area in the temporo-parietal (Hosseini et al., 2013; Beelen et al., 2019) areas, as well as the frontal cortex (Hosseini et al., 2013; Płoński et al., 2017), has been associated with dyslexia. Thus, we were interested in finding out, if interindividual differences in the surface area of these regions also predict future literacy skills.

## Materials and methods

### Study participants

Eighty-six German-speaking preliterate children were recruited in their last year of kindergarten on a voluntary basis throughout the city of Berlin. Advertisements in newsletters, kindergartens, and social media platforms were the main recruitment channels. Initial screening ensured that participants had no history of neurological diseases and normal hearing and visual acuity. All participants scored above the 85th percentile on the non-verbal part of the German adaption of the Wechsler Intelligence Scale for Children (WISC-IV; Petermann and Petermann, 2011) tested in the second grade of primary school. Furthermore, children were screened for reading expertise to ensure true preliteracy using a custom-made screening test (see Supplementary material and Liebig et al., 2021 for a detailed description). Both parents and children were carefully briefed about the longitudinal study design and the functional magnetic resonance imaging (MRI) constraints. Parents gave written informed consent and received compensation for their travels. All children gave their informed consent to participate in the study and were given age-appropriate education gifts. The Ethics Committee of the German Association for Psychology (DGPs) approved the study.

At the first appointment, nine children refused to participate in the MRI training session (mock-scanner) and were thus excluded from the study. All children who successfully participated in the actual functional MRI session were reinvited 2 years later at the end of the second grade. Ten participants could not be reinvited for the second appointment, and two children had to repeat the second grade and were tested 1 year later. Twenty-five children were excluded from the T1-weighted image analysis pipeline due to poor image quality or insufficient cortex reconstruction (discussed in section “T1-weighted imaging analysis”). The final sample consisted of 42 children, as summarized in Table 1. Seven of them had at least one first or second-degree relative with diagnosed developmental dyslexia stipulated by a parental questionnaire (Landerl and Moll, 2010).

**TABLE 1:** Demographic and psychometric information of the final pediatric sample before (TP1) and after literacy acquisition (TP2).

Descriptive data	Test	Raw scores (mean ± SD)	Range of raw scores	Percentile ranks (mean ± SD)
**Demographic information**				
Age at TP1		5.58 ± 0.48	5.01–6.09	
Age at TP2		8.25 ± 0.53	7.41–8.92	
Female/male		24/18		
Family history of dyslexia		7		
Right-handed/left-handed		38/4		
Monolingual/bilingual		37/5		
Non-verbal intelligence at TP1	CPM	23.26 ± 5.37	13–35	
Non-verbal intelligence at TP2	WISC	115.48 ± 12.71	90–147	
Dyslexia at TP2		10		
**Literacy precursor abilities (at TP1)**				
Rapid naming	BISC	14.81 ± 3.79	5–20	n.a.
Phonological awareness	BISC	36.17 ± 3.41	24–40	n.a.
**Literacy abilities (at TP2)**				
Reading fluency	SLRT-II	36.76 ± 15.69	6–71	52.95 ± 34.52 ^lp^ 54.94 ± 33.91 ^hp^
Reading comprehension	ELFE 1-6	54.00 ± 22.71	6–90	49.49 ± 32.14
Spelling accuracy	DERET 1-2 +	17.36 ± 11.56	1–50	40.24 ± 30.74
Literacy ability	SLRT-II, ELFE 1-6, DERET 1-2 +	0.00 ± 0.98	−2.60 to 1.84	n.a.

Dyslexia was defined as performance below the 16th percentile rank of the reference population in either spelling accuracy or real word reading fluency or in both based on Kuhl et al.’s (2020) classification criteria. CPM, Colored Progressive Matrices; WISC, Wechsler Intelligence Scale for Children; BISC, Bielefelder Screening zur Früherkennung von Lese-Rechtschreibschwierigkeiten; SLRT-II, Salzburger Lese- und Rechtschreibtest; ELFE 1-6, Ein Leseverständnistest für Erst- bis Sechstklässler; DERET 1-2+, Deutscher Rechtschreibtest für das erste und zweite Schuljahr; n.a., age-standardized scores are not available for subtests and literacy ability overall; lp, percentile lower boundary; hp, percentile higher boundary.

### Psychometric assessment

This study applied an extensive battery of psychometric testing at the two aforementioned developmental time points. Only tests used for the analyses of the present paper are described in this section [for a detailed description of all assessments, see Liebig et al. (2021) and the Supplementary material).

At the first assessment (TP1), i.e., prior to reading acquisition, PA and RAN were assessed with the *Bielefelder Screening zur Früherkennung von Lese-Rechtschreibschwierigkeiten* (BISC; Jansen, 2002). PA was calculated using several subtests of the BISC: syllable segmentation, rhyme recognition, word synthetization, and sound-to-word comparisons. RAN was operationalized by the time needed to name the color of objects printed in black and white and in an incongruent color. Errors made were sanctioned with a penalty time, i.e., incorrect responses were penalized with a longer reaction time. Non-verbal intelligence was measured using the *Raven’s Colored Progressive Matrices* (CPM; Raven and Court, 1998).

At the second assessment (TP2), i.e., after 2 years of schooling, children were tested on reading fluency and accuracy using two subtests of the *Salzburger Lese- und Rechtschreibtest* (SLRT-II) that focused on word and pseudoword reading speed and accuracy (Moll and Landerl, 2010). Reading comprehension was quantified using the *Ein Leseverständnistest für Erst- bis Sechstklässler* (ELFE 1-6; Lenhard and Schneider, 2006). This test captures reading comprehension on three levels with increasing complexity: word comprehension (decoding and synthesis), sentence comprehension (understanding of syntax), and text comprehension (understanding information and drawing conclusions). Spelling accuracy was assessed by writing from dictation using continuous text and gapped sentences using the German spelling test *Deutscher Rechtschreibtest für das Erste und Zweite Schuljahr* (DERET 1-2+; Stock and Schneider, 2008). Descriptive statistics for these psychometric tests are provided in Table 1.

### T1-weighted magnetic resonance image acquisition

T1-weighted images were acquired at TP1, i.e., at the end of kindergarten. A few days before the actual image acquisition, children had undergone a training session at the Max Planck Institute for Human Development Berlin using a mock scanner to familiarize them with the MRI procedure. The actual MRI session took place at the Centre for Cognitive Neuroscience Berlin (CCNB). Both sessions were adapted for young children. Their heads were cushioned with foam to ensure head stability and comfort, and age-appropriate earplugs were provided to attenuate scanner noise. Whole-brain anatomical images were gathered for each participant on a 3.0 Tesla Magnetom MRI system (Siemens Healthineers, Erlangen, Germany), equipped with a 12-channel head coil (repetition time: 2,000 ms, echo time: 30 ms, flip angle = 70°, 176 sagittal sections, voxel size: 2 mm × 2 mm × 2 mm, and field of view: 256 × 256 voxel matrix). Acquisition of the T1-weighted images followed a brief experiment in the scanner (described in Liebig et al., 2021) and lasted 4.5 min. During this time, a child-friendly video was played.

### T1-weighted imaging analysis

First, the T1-weighted images were visually inspected by two independent raters using Freeview 3.0, FreeSurfer’s visualization tool (Fischl, 2012), and MANGO 4.1, a multi-image analysis graphical user interface (Lancaster and Martinez, 2006). Additionally, image quality was assessed using the Computational Anatomical Toolbox (CAT) 12, an extension to the statistical parametric mapping (SPM) 12 software (Welcome Department of Cognitive Neurology; Ashburner et al., 2021). Fourteen participants were excluded due to severe ringing and blurring artifacts in the MRI scans caused by head motion. Children with moderate rigid body movement were marked and treated with special care in the subsequent visual inspection step, i.e., after reconstructing the surfaces during preprocessing. The cortices of eleven children were insufficiently reconstructed and excluded from subsequent analyses.

A fully automated pipeline of the FreeSurfer 7.1.1 software package (Fischl, 2012) was utilized to preprocess the T1-weighted MRI scans, which included removal of non-brain tissues, transformation, and intensity normalization, segmentation of white and deep gray matter, correction of topological errors, and reconstruction of the cortical surface. Surface area and cortical volume were extracted from the T1-weighted image. In FreeSurfer, surface area is quantified as the sum of the areas of adjacent triangle faces on the surface mesh, computed in each participant’s native space, allowing for individual variations in the area of each triangle. Cortical volume is defined as the amount of gray matter between the gray/white and pial boundary. These features were modeled for each hemisphere separately.

After completing all preprocessing steps, the segmentation of each participant’s cortex was visually inspected in Freeview 3.0 (Fischl, 2012) to ensure accurate classification of gray--white matter boundaries, correct skull stripping, and true separation between brain and non-brain matter. All surfaces were checked and edited in the coronal, sagittal, and axial planes to ensure optimal results. All edits strictly followed the guidelines provided by FreeSurfer.^1^ The editor was blind to the participants’ degrees of literacy. Subsequently, the adjusted images were reprocessed *via* the automated reconstruction pipeline and checked a second time for accurate reconstruction by the editor.

Next, the three-dimensional lGI proposed by Schaer et al. (2008) was computed in FreeSurfer and the Image Processing Toolbox of Matrix Laboratory (MATLAB) 2020b (The Math Works Inc, 2020) to measure the regional folding of the cortex using a spherical kernel of 25 mm at each vertex. Compared to other metrics of cortical folding such as curvature, sulcal depth measurement, and the classical two-dimensional gyrification index (Zilles et al., 1988), the lGI takes the inherent three-dimensional nature of the cortical surface into account and makes it robust against slice orientation and the presence of buried sulci. The automated construction of the lGI was validated against manual measurement and manifested as a reliable measure of gyrification (Schaer et al., 2012).

For the subsequent region of interest (ROI) analyses, eight left-hemispheric ROIs spanning all three reading streams were selected *a priori* based on the functional meta-analysis of Richlan et al. (2009) and previous research in children (Płoński et al., 2017; Beelen et al., 2019; Perdue et al., 2020). For the ventral stream, these included (1) the fusiform gyrus, (2) the inferior temporal gyrus, and (3) the middle temporal gyrus; the dorsal stream was represented by (4) the superior temporal gyrus, (5) the inferior parietal cortex, consisting of the inferior parietal and the angular gyrus, and (6) the supramarginal gyrus; finally, (7) the insular cortex, and (8) the inferior frontal gyrus, a result of combining pars opercularis and pars triangularis, linked to the frontal stream were selected. ROIs were taken from the Desikan-Killiany atlas, which subdivides the cortex into 34 gyral regions based on curvature and sulcal information on the inflated cortex for each hemisphere (Desikan et al., 2006). This automatic labeling has been discussed as having higher accuracy than manual parcelation (Desikan et al., 2006). Next, the mean metrics for all ROIs were extracted from the FreeSurfer output and imported into MATLAB. Although the FreeSurfer average participant is adult-based, it is frequently used in pediatric samples, and surface-based registration has been validated in children ages 1–11 with good alignment of cortical landmarks (Ghosh et al., 2010). No smoothing was applied to the lGI data as it is already intrinsically smoothed on the individual level as defined by the algorithms employed during the lGI procedure (averaging across a 25-diameter circle). Surface area and cortical thickness metrics were smoothed at full width half maximum (FWHM) of 20 mm to approximate the intrinsic smoothing of the lGI algorithms and increase the signal-to-noise ratio (mri_surf2surf). In contrast to volumetric smoothing, surface-based smoothing only averages data from nearby vertices on the cortical surface, preventing the merging of signals from different tissue types and resulting in higher spatial specificity (Greve and Fischl, 2018).

### Statistical analyses

Demographic and psychometric data were assessed in MATLAB 2020b. Literacy ability was calculated as the mean of reading fluency of words and pseudowords (SLRT-II), reading comprehension of words, sentences, and text (ELFE 1-6), and spelling accuracy scores from dictation (DERET 1-2+) to match other attempts of reading predictions in German-speaking samples (e.g., Kuhl et al., 2020). Instead of age-normed standard scores, raw scores for all psychometric measures were utilized as we were interested in the within-subject association between measures at TP1 and TP2 and not in comparisons between peers.

A CV elastic net linear regression (Zou and Hastie, 2005) was used to perform a variable selection of the best preliterate brain and behavioral predictors of literacy ability, as introduced above. Furthermore, previous studies have shown that there may be subtle differences in literacy skills between females and males as well as an association with non-verbal intelligence (Flannery et al., 2000; Rutter et al., 2004; Liederman et al., 2005; Cotton and Crewther, 2009; Halpern, 2013; Quinn and Wagner, 2015). Therefore, sex and non-verbal intelligence measured at TP1 were added as additional prediction variables to the model. All analyses steps outlined below were implemented in R 4.1.2 (R Core Team, 2021) using the packages caret 6.0-90 (Kuhn, 2015) and glmnet 4.1-3 (Friedman et al., 2010).

Regularized analysis methods such as elastic net regressions are better suited for handling neuroimaging data than classical linear regression models because of their superiority in dealing with inter-correlated predictors (Carroll et al., 2009). In regularized linear models, a penalty term is added to the least-squares objective function (Hoerl and Kennard, 1970). The amount of penalization is governed by smoothing parameters. The penalty, in turn, controls the bias-variance trade-off by reducing variance at the cost of deliberately introducing some bias into the resulting estimators (Hastie et al., 2009). The elastic net penalty (Zou and Hastie, 2005) combines the power of a least-absolute-shrinkage-and-selection-operator (LASSO) regularization (Tibshirani, 1996) to select relevant variables in the model, i.e., set the weights of certain coefficients to zero, with a Ridge penalty (Hoerl and Kennard, 1970), which takes correlation between prediction variables better into account (Cho et al., 2010). Thus, highly correlated predictors are retained or discarded from the model as variables, making it an ideal regression approach for brain data with high ratios of features to cases (Zou and Hastie, 2005; Whelan and Garavan, 2014).

The elastic net aims at minimizing the following loss function:

$$L_{e\,n\,e\,t}\,\left(\hat{\beta }\right)=\frac{\sum_{i=1}^{n}{\left(y_{i}-x_{i}^{T}\,\hat{\beta }\right)}^{2}}{2\,n}+\lambda \,\left(\frac{1-\alpha }{2}\,\sum_{j=1}^{m}{\hat{\beta }}_{j}^{2}+\alpha \,\sum_{j=1}^{m}\left|{\hat{\beta }}_{j}\right|\right),$$

where $\sum_{j=1}^{m}{\hat{\beta }}_{j}^{2}$ represents the LASSO (or L1), and $\sum_{j=1}^{m}\left|{\hat{\beta }}_{j}\right|$ the Ridge (or L2) penalty, λ is the tuning parameter that determines the weight of the composite regularization term, i.e., the bias-variance trade-off, and α the hyperparameter that controls the balance between the two types of penalties. The former parameter ranges from 0 to infinity, with λ = 0 resulting in the ordinary least squares solution due to eliminating the penalty from the equation. The latter hyperparameter may take on values from 0 to 1. If α = 0, the regression is identical to the Ridge regression; if α = 1, the L2 term cancels out, and the penalty corresponds to the LASSO penalty.

**FIGURE 1:**
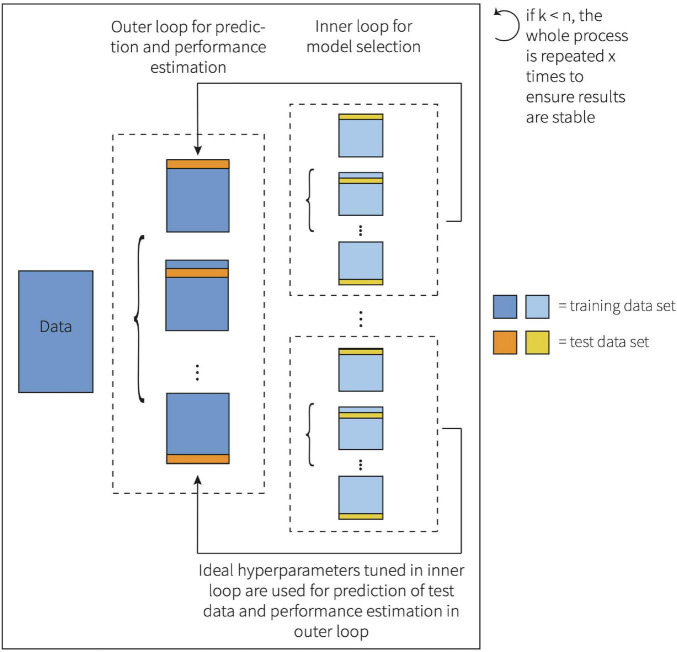
Representation of the nested *k*-fold cross-validation framework.

Here, both α and λ were estimated within the inner loop of a nested leave-one-out cross-validation (LOOCV). LOOCV is the most extreme form of *k*-fold CV (Allen, 1974; Stone, 1974; Geisser, 1975). When using LOOCV, the number of folds equals the number of observations (*k* = *n*), which is especially valuable when the sample size is small (Allen, 1974; Stone, 1974). A nested LOOCV method was chosen to avoid a biased, overly optimistic estimate of the true generalization error, which may be the case if observations are part of both the training and test dataset (Varma and Simon, 2006). This framework is visualized in Figure 1.

The best model in the inner loop was selected based on the lowest prediction error, quantified by the root mean squared error (RMSE). The resulting model was then utilized in the outer loop to predict literacy ability. In turn, the vector of predicted test observations over iterations was entered into several formulas to calculate the following goodness-of-fit measures: the mean absolute error (MAE), the RMSE, and *R*^2^ based on the test set observations. Top prediction variables were identified based on their variable importance as calculated by caret (Kuhn, 2015).

An ordinary least squares linear regression was computed using only the two cognitive-linguistic skills, i.e., PA and RAN. The resulting RMSE was then compared to the RMSE from the elastic net model to determine if adding the gray matter macrostructural prediction variables and covariates would improve the prediction of literacy ability.

For comparison, the prediction model was recomputed using a nested 10-fold CV procedure. This process was repeated 50 times to enhance the estimate of the true unknown underlying mean model performance by fitting and evaluating more models and thereby controlling for potential biases caused by the pseudorandom split of the data (Vehtari et al., 2017).

Frequently, estimated total intracranial volume (eTIV) is used as a covariate in similar research paradigms. Therefore, the LOOCV model was recomputed with eTIV as an additional prediction variable. However, this variable was not part of the final model because past research has suggested that controlling for eTIV may overcorrect for differences in head volume and may reduce individual differences in continuous regression approaches (Westman et al., 2013; Wierenga et al., 2014). Due to this incongruity, we decided to focus on the more parsimonious model, in line with Occam’s razor (Blumer et al., 1987).

Furthermore, partial correlations between the gray matter features within the ROIs were calculated to investigate the associations between the different indices. The resulting *p*-values were Holm–Bonferroni family-wise error corrected. A correlational whole-brain analysis was conducted to identify potential areas associated with literacy ability but was not captured by the selected ROIs. The methodology and results are described in Supplementary material.

## Results

**FIGURE 2:**
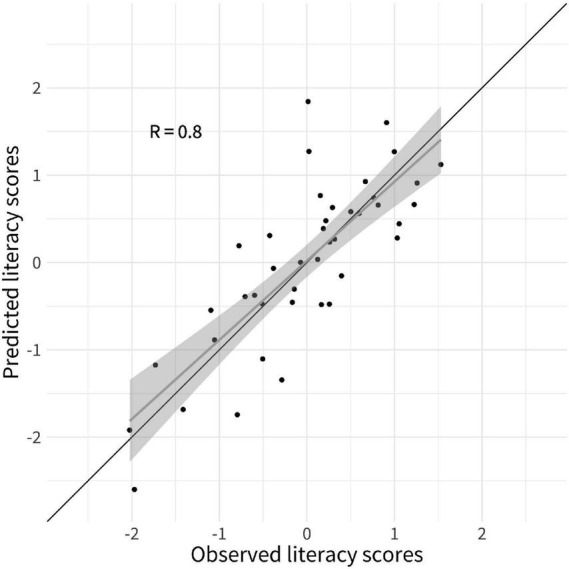
Predicted literacy ability from leave-one-out cross-validated (LOOCV) elastic net regression model. Scatterplots depict the observed literacy ability (*y*-axis) by predicted literacy skills (*x*-axis). The black line represents the line of identity; the gray line is the regression line of literacy ability on predicted literacy, with the shaded area representing a 95% pointwise confidence interval.

A LOOCV elastic net regression model was computed to identify the behavioral and gray matter features measured at a preliterate age that were the strongest predictors of literacy ability 2 years later. The predictive strength of literacy ability was improved when gray matter macrostructural features were added as prediction variables on the top of the cognitive-linguistic preliterate skills variables, i.e., PA and RAN. More precisely, the RMSE of 0.82 decreased by 29% to 0.58 in the elastic net model that included the gray matter macrostructural features. The same elastic net regression produced highly accurate estimations of literacy ability as testified by a high correlation coefficient between predicted and observed values of *r* = 0.80 (see Figure 2).

On average, the LOOCV models were reduced to 20 prediction variables, i.e., dropping approximately eight predictors in each iteration. This was affected by a low mean value of lambda, $\bar{\lambda }$ = 0.01, and a high mean value of alpha, $\bar{\alpha }$ = 0.85, which led to a small weight of the composite penalty term with a stronger contribution of the LASSO penalty. The models were approximately stable over iterations, as indicated in Table 2.

**TABLE 2:** Tuned hyperparameters and selected non-zero coefficients of the leave-one-out (LOO-) and 10-fold cross-validated (CV) elastic net regressions.

	Alpha (mean ± SD)	Lambda (mean ± SD)	Number of non-zero coefficients (mode, range)
LOOCV	0.853 ± 0.328	0.012 ± 0.006	20, 16–27
10-fold CV	0.658 ± 0.392	0.022 ± 0.016	19, 13–27

The count of regression variables does not include the model’s intercept. Twenty-eight variables were entered into the model. SD, standard deviation.

The features with the greatest contribution to the prediction of literacy ability were lGI in the insular cortex and cortical volume in the fusiform gyrus. Additionally, lGI in the supramarginal and posterior inferior frontal gyrus and cortical volume in the inferior temporal gyrus were also important variables in predicting reading and writing skills. Both cognitive-linguistic prediction variables were in the final model, with PA ($\bar{b}=0.41$) explaining twice as much variance in literacy ability as RAN ($\bar{b}=0.18$). Moreover, sex contributed unique variance to the model ($\bar{b}=0.15$): Females showed a greater probability of slightly higher literacy scores than males. Non-verbal intelligence ($\bar{b}=0.01$) was excluded from the model in most iterations of the LOOCV procedure.

**FIGURE 3:**
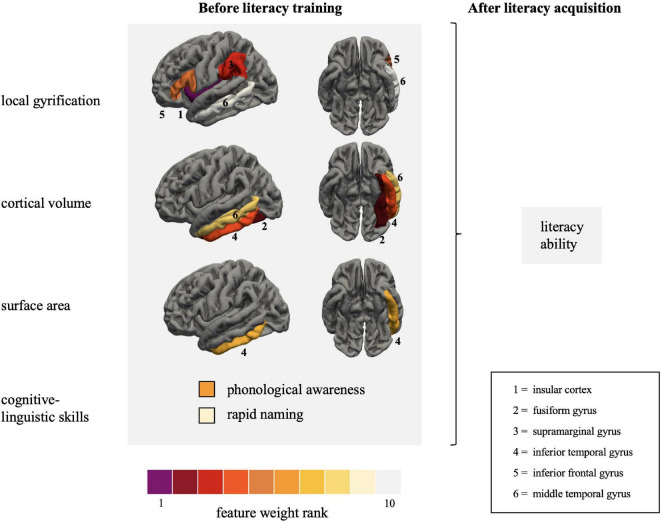
Visualization of the primary features collected at the end of kindergarten predicting literacy ability measured at the end of the second grade. Features with the greatest contribution to the prediction of literacy ability are coded purple and red. The regions are depicted on the left pial surface of the FreeSurfer template based on the Desikan-Killiany atlas (Desikan et al., 2006). Literacy ability is defined as the summary score of reading fluency, reading comprehension and spelling accuracy, measured with the *Salzburger Lese- und Rechtschreibtest*, *Ein Leseverständnistest für Erst- bis Sechstklässler*, and *Deutscher Rechtschreibtest für das erste und zweite Schuljahr*, respectively.

The 10-fold CV elastic net model revealed results comparable to the LOOCV model, as documented in Tables 2–4. A list of the central gray matter and behavioral prediction variables of the leave-one-out and 10-fold CV models is provided in Table 4 and visualized in Figure 3. A complete listing of all predictors is provided in Supplementary material. Model predictions, mean coefficients and feature ranks remained almost unchanged when intracranial volume was added as a covariate.

**TABLE 3:** Model performance of the leave-one-out (LOO-) and 10-fold cross-validated (CV) elastic net regressions.

	RMSE	MAE	*R* ^2^	*R*
LOOCV	0.575	0.459	0.652	0.807
10-fold CV	0.579	0.438	0.646	0.804

R, coefficient of determination; RMSE, root mean squared error; MAE, mean absolute error.

**TABLE 4:** The top ten prediction variables of literacy ability (mean coefficient >0.15) based on the leave-one-out (LOO) and 10-fold cross-validated (CV) elastic net linear regressions.

Rank	Selection frequency (LOOCV)	Mean coefficient		Gray matter feature or psychometric variable	Region of the left hemisphere
		LOOCV	10-fold CV		
1	76.19%	0.71	0.61	Local gyrification	Insular cortex
2	71.43%	0.69	0.59	Cortical volume	Fusiform gyrus
3	71.43%	−0.50	−0.43	Local gyrification	Supramarginal gyrus
4	64.29%	−0.47	−0.43	Cortical volume	Inferior temporal gyrus
5	57.14%	−0.40	−0.33	Local gyrification	Inferior frontal gyrus
6	59.52%	0.41	0.41	Phonological awareness	
7	76.19%	−0.24	−0.24	Surface area	Inferior temporal gyrus
8	61.90%	0.21	0.19	Cortical volume	Middle temporal gyrus
9	38.10%	0.18	0.18	Rapid naming	
10	35.71%	0.18	0.15	Local gyrification	Middle temporal gyrus

Predictors are listed according to their average rank. The rank displays the variable importance as defined by caret (Kuhn, 2015), i.e., how much unique variance of the response variable can be explained by this variable. Compared to the mean correlation coefficient, this metric is more stable against outlier models. The selection frequency shows how often the variable was chosen at this rank for the LOOCV regression. All prediction variables were standardized before being entered into the model.

Correlational analyses revealed a high, positive association of cortical volume and surface area across ROIs (*r* = 0.87–0.96, *p* < 0.001 Holm–Bonferroni corrected). In contrast, lGI correlated only low to moderately with cortical volume (*r* = 0.03–0.41) and surface area (*r* = 0.06–0.45).

## Discussion

The present study aimed to predict future literacy in preliterate children using a continuous sampling approach. We successfully applied a linear regression approach to predict future reading acquisition of children with measures gathered at the end of kindergarten (i.e., before formal reading instruction). The CV model captured individual differences in future literacy based on gray matter macrostructural features and cognitive-linguistic preliterate skills measured at the end of kindergarten. More specifically, the elastic net regularized linear regression models predicted approximately 65% of the variance in literacy 2 years later (Figure 2). Intriguingly, the top five features contributing to the prediction are part of the three major reading streams. These were lGI in the insular cortex, the inferior frontal gyrus, the supramarginal gyrus, and cortical volume in the fusiform gyrus and inferior temporal gyrus. This pattern emphasizes that reading readiness in all crucial parts of the large-scale reading network differs among children and that these individual variations significantly impact reading acquisition.

### Individualized prediction of future literacy in cross-validated frameworks

The identification and validation of biomarkers for the early detection of children at risk of developing reading difficulties have been of major interest over the past years. A better knowledge of the prerequisites facilitating or hampering reading acquisition improves our understanding of the multifaceted learning process and forms the basis for developing specific preventive treatment strategies. Recently, it has been advocated to replace the traditional correlational approach with prediction frameworks to generalize the observed patterns to independent data sets (Gabrieli et al., 2015; Dubois and Adolphs, 2016). In line with this, we applied CV regression models to assess the composition of neural and behavioral markers of future reading acquisition. We were able to predict future literacy with high precision. Compared to classical linear regressions, the algorithm prevents over-fitting by adding additional constraints to the model. Automatic feature selection in the training phase results in sparse predictive models, making it an optimal algorithm for neuroimaging, characterized by many features and small sample sizes (Cui and Gong, 2018). Computationally, our results replicate and thus further demonstrate the effectiveness of elastic net penalized linear regressions for gray matter-based reading prediction as reported by Cui et al. (2018) for a large sample size (*N* = 870). We further generalize its suitability to the longitudinal prediction of literacy skills based on macrostructural and behavioral information. Similar to Cui et al. (2018), applying the previously built models to new cohorts of preliterate children in cross-linguistic studies would be interesting. If the biomarkers identified here could be replicated across languages, this would strengthen the generality of the approach and the validity of the predictors thus making it possible to test for orthography-specific effects.

### The predictive power of reading streams

The three central reading streams did not equally contribute to predicting literacy. Compared to the ventral and frontal streams, individual variations in the macrostructural features linked to the dorsal stream played a minor role. Indeed, out of three *a priori* defined ROIs associated with the dorsal stream, only the supramarginal gyrus was consistently selected during the CV prediction. Interestingly, Linkersdörfer et al. (2012) identified a link between gray matter reduction and functional underactivation in the supramarginal gyrus in dyslexia. The present findings strongly suggest that individual variations in the supramarginal gyrus apply to impaired reading and affect future literacy in a wide range of typically developing children. In a previous study, we examined possible early neurofunctional literacy markers in the same cohort of children (Liebig et al., 2021). We observed a correlation between RAN and neural functioning in the supramarginal gyrus. Likewise, a cluster in the angular gyrus, extending to the supramarginal gyrus, predicted future reading fluency. The latter, however, did not survive additional rigorous correction for the number of regression models (Liebig et al., 2021). Taken together, the present results converge with our previous findings to suggest that variations in both the functional and structural architecture of the supramarginal gyrus might be a promising biomarker for predicting future reading acquisition.

In contrast, neither the superior temporal gyrus nor the inferior parietal cortex explained significant amounts of unique variance in literacy skills. On the one hand, the subordinate role of the dorsal stream stands in contrast with the classical model of reading acquisition, according to which initial decoding relies on the dorsal stream. In contrast, parallel automatized word recognition relies on the ventral stream and emerges only later during reading acquisition (Pugh et al., 2000, 2013). On the other hand, recent functional (Kronbichler et al., 2007; Price and Devlin, 2011; Richlan et al., 2011; Liebig et al., 2017) and structural (Richlan et al., 2013; Williams et al., 2018) brain imaging studies on reading acquisition strongly emphasize the crucial role of the ventral stream not only in beginning readers but already in preliterate children (Hoeft et al., 2011; Liebig et al., 2021). The fact that all *a priori* defined regions of the ventral stream were reliably selected during the prediction iterations supports the idea that neuroanatomical characteristics of the ventral occipito-temporal cortex are essential determinants of successful reading acquisition. This finding does not question the general importance of the dorsal stream for initial decoding (Martin et al., 2015; Zhou et al., 2016; Liebig et al., 2017; Braun et al., 2019), but it raises the question of whether the transition from serial decoding to rapid parallel access to written word forms (automatization) requires the integrity of specific neuroanatomical properties related to the ventral stream that can be assessed even prior to reading.

Our results thus extend the fast-growing knowledge about the early importance of the ventral stream in several ways: Firstly, macrostructural features of the ventral stream do not only distinguish between children and adults with and without dyslexia (Linkersdörfer et al., 2012; Richlan et al., 2013) but individual differences in the morphology of gray matter features significantly contribute to the individualized prediction of future reading acquisition. This has been observed in the functional data of Liebig et al. (2021), who showed that neural activity in the ventral stream correlated with RAN and predicted future reading fluency in the same cohort of children. Taken together, we observed highly similar relationships in the same cohort of children on the functional and structural levels when applying different computational approaches (CV prediction vs. classical correlational analysis) both on the whole-brain level and in an ROI-based study. This convergence across the two studies clearly supports the plausibility of our effects. Similarly, the results converge with a recent finding to suggest that increased neural plasticity of temporo-parietal regions in emergent readers supports reading acquisition (Phan et al., 2021) paving the way for early identification and targeted intervention of children at-risk of encountering difficulties during reading development.

Like the ventral stream, all parts of the frontal stream significantly contributed to the prediction models. This result is in line with the interactive account of reading (Price and Devlin, 2011), according to which reading acquisition is marked by top-down influences from frontal to ventral occipito-temporal regions. Hence, individual differences in the gyrification pattern of the frontal stream should be seen in concert with gray matter features in the ventral stream.

### Gray matter macrostructural features underlying future literacy

In the present study, we compared different macrostructural indices (lGI, cortical volume, and surface area) to evaluate their suitability for the individualized prediction of literacy. We found that lGI and cortical volume had better predictive power than surface area. Regional specificities seem to drive the individualized prediction when looking at the distributional pattern. Cortical volume was the decisive feature in the ventral stream, whereas the lGI was the strongest feature in the frontal and dorsal stream, which makes it a promising macrostructural feature in relation to reading. Computationally, the lGI allows a more reliable calculation of the cortical folding than previous measures because it utilizes three-dimensional surface properties to fully capture the patterns of the cortical mantle (Schaer et al., 2008). Using lGI as a measure, it has already been shown that developmental dyslexics exhibited a thinner and more gyrified left occipito-temporal cortex (Williams et al., 2018) and a more gyrified primary auditory cortex (Kuhl et al., 2020). The present results suggest that the lGI is also suitable to detect subtle individual differences in continuous sampling. However, future studies need to replicate and thus validate the suitability of lGI in relation to literacy skills.

The considerable importance of cortical volume in the ventral stream is in line with previous results. Several meta-analyses confirm that cortical volume in the ventral occipito-temporal cortex distinguish children and adults with and without dyslexia (Linkersdörfer et al., 2012; Richlan et al., 2013) and is generally associated with reading skills (Eckert et al., 2016; Skeide et al., 2016).

Surface area only played a minor role in the individualized prediction of literacy ability, which might be explained by the high correlation of surface area and cortical volume across regions of interest (*r* = 0.87–0.96). This is in line with the notion that cortical volume is the product of cortical thickness and surface area (Winkler et al., 2010). Instead, lGI and cortical volume were only moderately correlated (*r* = 0.03–0.42). When aiming to capture different aspects of variance in the gray matter, it might thus be advisable to focus on not too strongly correlated features and integrate these into the prediction models.

In the present study, we observe both negative and positive relationships between literacy and the macrostructural features depending on the ROIs. The associations can be characterized as follows: previously, different macrostructural features, i.e., cortical volume and thickness of the supramarginal gyrus, were reliably associated with reading skills. Both positive relationships between cortical volume (Jednoróg et al., 2015; Xia et al., 2018) and longitudinal volume reductions (Houston et al., 2014; Linkersdörfer et al., 2014) were associated with reading skills. In the present study, we observed a negative relationship between literacy and the lGI in the supramarginal gyrus, which was frequently selected as the third most important predictor of literacy. This finding strongly suggests that the gyrification pattern in the dorsal stream also affects literacy.

We observed both positive and negative associations in the ventral stream: while cortical volume in the fusiform gyrus and the middle temporal gyrus was positively associated with future literacy, both cortical volume and surface area exhibited a negative relationship with reading and writing. The positive association partly contradicts previous findings in children (Simon et al., 2013; Skeide et al., 2016). However, the operationalization of literacy and the age of the samples differ substantially from the present study. While we examined literacy ability on different levels to better account for this multifaceted nature of reading, Simon et al. (2013) and Skeide et al. (2016) focused on reading speed. However, positive relationships have been reported when also looking at cortical thickness (Xia et al., 2018). In sum, there is cumulative evidence that different regions of the ventral occipito-temporal cortex crucially relate to individual differences in reading ability and distinguish between children with and without dyslexia (Płoński et al., 2017; Beelen et al., 2019). In the present study, the cortical volume of the fusiform gyrus and the inferior temporal gyrus could explain twice as much unique variance as the two literacy precursory skills. This robust finding aligns with the increasingly recognized importance of the ventral stream in the first steps of reading acquisition (e.g., Hoeft et al., 2011; Liebig et al., 2021).

Local gyrification in the frontal stream also showed both directions, i.e., positive in the insular cortex and negative in the inferior frontal gyrus. Although the insular cortex is an integral part of the language and reading network (Price, 2012) its macrostructural features have seldomly been examined concerning reading acquisition. The lGI of the insular cortex was selected as the strongest predictor of future literacy. The insular cortex has previously been associated with diverse aspects of language and reading (Price, 2012). Most interesting for the present study, the insula might be a crucial part of the phonological network in reading acquisition, which is delayed in children with developmental dyslexia (Łuniewska et al., 2019). Similarly, the insula seems to be more strongly involved in pre-readers compared to readers emphasizing its importance during the first steps of reading acquisition (Monzalvo and Dehaene-Lambertz, 2013; Chyl et al., 2018). With the present results, we provide first evidence that the gyral folding pattern of the insular cortex might be a promising early biomarker of future literacy acquisition in native German-speaking children. However, future research needs to refine this ample evidence and disentangle the contribution of different aspects of gray matter morphology and possible sensitive phases of cortical plasticity. The idea of the interaction of neural plasticity and reading acquisition was recently endorsed by a structural neuroimaging study showing a gray matter volume increase in decisive regions of the ventral and dorsal reading network during the earliest phases of reading acquisition (Phan et al., 2021). Whether this holds for the insular cortex as well needs to be tackled in future research.

In general, the observed pattern of regional-specific directions of the relationships found in the same cohort of participants is in line with previous continuous approaches (Jednoróg et al., 2015; Skeide et al., 2016).

### Cognitive-linguistic preliteracy skills

The cognitive-linguistic preliterate skills were among the top ten features that were frequently selected in the CV approach with PA being a stronger predictor of literacy than RAN. This pattern does not entirely align with previous behavioral results in German-speaking children. It has been reported that preliterate RAN reliably predicts reading while PA only becomes significant in beginning readers (Landerl et al., 2019). However, in the present study, we combine neural and behavioral information in a CV predictive framework and operationalized literacy on different levels of complexity, which might have led to the observed differences. Our results suggest that a combination of both behavioral and macrostructural features makes it possible to predict reading outcomes with high accuracy even before the onset of literacy instruction.

## Limitations and conclusions

Prior work linking brain anatomy to reading ability was primarily based on groups with and without dyslexia (Linkersdörfer et al., 2012; Richlan et al., 2013). Only a few studies investigated this link with a continuous approach (e.g., Houston et al., 2014; Jednoróg et al., 2015; Torre and Eden, 2019). However, developmental trajectories might differ between individuals within each group of typical and dyslexic readers (Chyl et al., 2021). Thus, continuous sampling and group-based approaches should be combined to track both group differences and interindividual differences. Such an integrated approach allows identifying both general neural makers applicable to the entire range of reading acquisition and abnormal patterns related explicitly to impaired reading.

Furthermore, examining very young children in the MRI scanner led to a greater motion and thus lower image quality than studies with older children. However, we thoroughly controlled the images and applied rather strict dropout criteria to control for the pitfalls of pediatric neuroimaging. From a theoretical perspective, we only provide preliminary insights into the prerequisites of reading acquisition. We systematically targeted possible predictors of future literacy by comparing several macrostructural and behavioral measures. However, even in a CV predictive framework, in which the number of features may expand the number of observations, the maximal number of features has to be limited when aiming to obtain interpretable results. Thus, we utilized an ROI approach rather than a whole-brain analysis and limited the number of gray matter structural features. *A priori* selection of regions and features may risk overseeing relationships beyond the targeted areas and features.

Apart from these limitations, the present study is a further step in applying CV models to examine biomarkers of typical reading acquisition in pediatric neuroimaging. Individual variations in several macrostructural gray matter features in crucial parts of the large-scale left-hemispheric reading network predicted literacy skills 2 years later with high precision. In the predictive framework, the ventral and frontal streams showed considerable importance. Thus, from a theoretical perspective, our results support recent arguments about the importance of the ventral stream in reading acquisition (Hoeft et al., 2011; Richlan, 2012; Liebig et al., 2017) in concert with top-down modulation of the frontal stream (Price and Devlin, 2011).

From a clinical perspective, the present results might also have implications for education and therapy. We provide evidence that children might come to the task of learning to read with different initial conditions at the neuroanatomical and behavioral level that might well impact how quickly and efficiently they will be able to learn at school (see also Liebig et al., 2021). With the increasing number of longitudinal structural and functional studies conducted at the end of kindergarten pointing in the same direction (Chyl et al., 2021), our results clearly favor an early diagnosis of future reading difficulties. Structural neuroimaging might be a promising tool, given that gray matter features are far easier to acquire than functional neuroimaging. Firstly, no task is required and thus, imaging time notably drops compared to functional imaging. Secondly, the requirements put upon the children in terms of attention and compliance decrease substantially in structural neuroimaging, making the features gathered even more objective. Thus, structural imaging seems more feasible in a clinical routine and daily practice than functional neuroimaging. Notably, we observed a substantial overlap of those regions contributing to the prediction in the structural and functional analysis in the same cohort of children (see Liebig et al., 2021, for the functional analysis), emphasizing the potential of gray matter features to be become early biomarkers of normal and impaired reading development.

## Data availability statement

The raw data supporting the conclusions of this article will be made available by the authors, without undue reservation.

## Ethics statement

The studies involving human participants were reviewed and approved by the German Association for Psychology. Written informed consent to participate in this study was provided by the participants’ legal guardian/next of kin.

## Author contributions

JL, EF, JZ, and AJ designed the study. MBe analyzed the data with advice from JL and TS. MBe and JL drafted the manuscript. All authors contributed to the final version of the article.

## Footnotes

1
http://surfer.nmr.mgh.harvard.edu/fswiki/FsTutorial/TroubleshootingData
