# Supplementary material for: Structural gray matter features and behavioral preliterate skills predict future literacy – A machine learning approach
Source: Front Neurosci. 2022 Sep 29;16:920150. doi: 10.3389/fnins.2022.920150 (PMC9558903; doi:10.3389/fnins.2022.920150)
Supplement: Supplementary file 1 [file Data_Sheet_1.docx]

**Supplementary Materials**

**1 Psychometric Assessment**

**Timepoint 1 (end of kindergarten)**

Custom-made screening test that assessed basic letter knowledge (e.g., *a, d*), picture-word matching of highly frequent words (e.g., *ball, cow*), syllable reading (e.g., *pa, som*), decoding of phonotactically valid pseudowords (e.g., *Muma, Ticht*) and word reading (e.g., *father, evening*).

**Timepoint 2 (after two years of formal reading instruction)**

We added measures of verbal development (scope of lexicon) and non-verbal working memory (digit span) also using the *Wechsler Intelligence Scale for Children* (WISC-IV; Petermann & Petermann, 2014).

Reading speed and comprehension on the sentence level were evaluated using a plausibility judgement test (Salzburger Lese-Screening für die Klassenstufen 1-4; SLS; Mayringer & Wimmer, 2008). The age-normed SLS score is a combined score capturing reading speed and accuracy with a scaling that is equal to the intelligence quotient.

Certain aspects of Phonological awareness were tested (Basiskompetenzen für Lese-Rechtschreibleistungen: BAKO 1-4; Ein Test zur Erfassung der Phonologischen Bewusstheit vom ersten bis vierten Grundschuljahr; Stock et al., 2003; subtests: phoneme transition, sound categorization, vocal length).

We also screened children’s exposure to books *(K-TRT: Kinder Titelrekognitionstest; Schroeder et al., 2016)*.

**2 Elastic Net Regression Analysis**


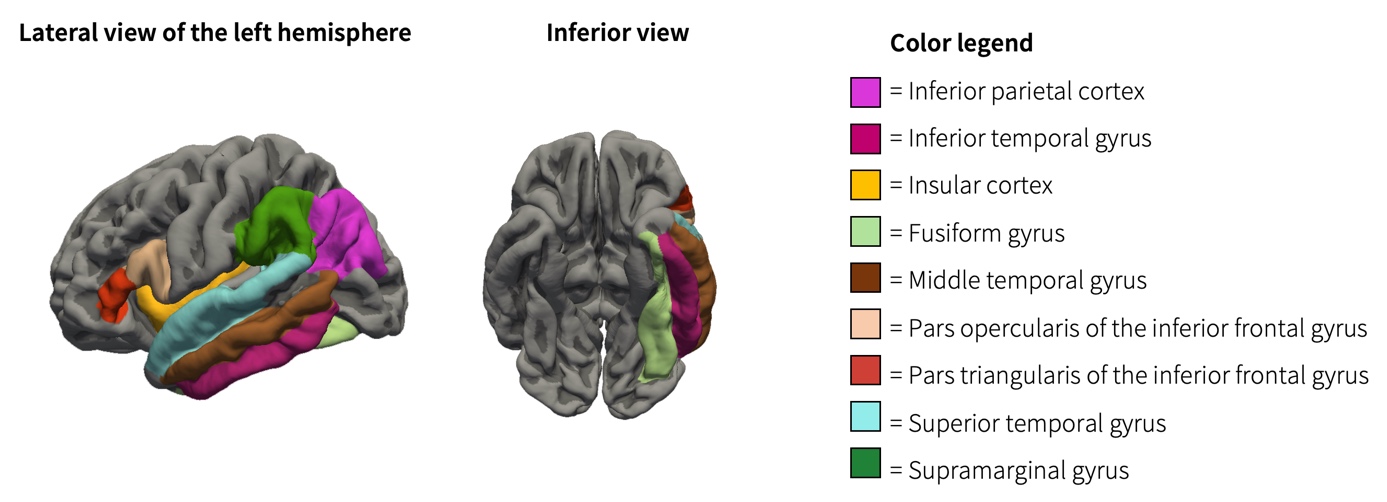


**Supplementary Figure 1.** The regions of interest based on the Desikan-Killiany Atlas (Desikan *et al.*, 2006) visualized on the left pial surface of the FreeSurfer template. The pars opercularis and pars triangularis of the inferior frontal gyrus were merged for analyses.

| Mean coefficient | | Grey matter feature or psychometric variable | Region of the left hemisphere |
| --- | --- | --- | --- |
| LOOCV | 10-fold CV |  |  |
| 0.71 | 0.61 | Local gyrification | Insular cortex |
| 0.69 | 0.59 | Cortical volume | Fusiform gyrus |
| - 0.50 | - 0.43 | Local gyrification | Supramarginal gyrus |
| - 0.47 | - 0.43 | Cortical volume | Inferior temporal volume |
| 0.41 | 0.41 | Phonological awareness |  |
| - 0.40 | - 0.33 | Local gyrification | Inferior frontal gyrus |
| - 0.24 | - 0.24 | Surface area | Inferior temporal gyrus |
| 0.21 | 0.19 | Cortical volume | Middle temporal gyrus |
| 0.18 | 0.15 | Local gyrification | Middle temporal gyrus |
| 0.18 | 0.18 | Rapid naming |  |
| - 0.16 | - 0.17 | Surface area | Insular cortex |
| - 0.15 | - 0.13 | Cortical volume | Insular cortex |
| 0.15 | 0.14 | Sex |  |
| 0.13 | 0.12 | Local gyrification | Fusiform gyrus |
| - 0.09 | - 0.05 | Cortical volume | Supramarginal gyrus |
| 0.08 | 0.06 | Cortical volume | Inferior frontal gyrus |
| 0.03 | 0.08 | Surface area | Fusiform gyrus |
| - 0.03 | - 0.05 | Surface area | Superior temporal gyrus |
| 0.03 | 0.05 | Surface area | Inferior parietal cortex |
| - 0.02 | - 0.04 | Local gyrification | Inferior parietal cortex |
| 0.02 | 0.04 | Cortical volume | Superior temporal gyrus |
| - 0.01 | - 0.01 | Surface area | Supramarginal gyrus |
| 0.01 | 0.03 | Surface area | Inferior frontal gyrus |
| - 0.01 | - 0.01 | Local gyrification | Inferior temporal gyrus |
| 0.01 | 0.01 | Non-verbal intelligence |  |
| 0.00 | 0.01 | Surface area | Middle temporal gyrus |
| 0.00 | 0.00 | Local gyrification | Superior temporal gyrus |
| 0.00 | 0.00 | Cortical volume | Inferior parietal cortex |

**Supplementary Table 1.** All prediction variables of literacy ability based on the leave-one-out cross-validation (LOOCV) and 10-fold cross-validated (CV) elastic net linear regressions. Predictors are listed according to their mean correlation coefficient of the LOOCV procedure. All prediction variables were standardized before being entered into the model.

## 3 Vertex-Wise Whole-Brain Analysis

**3.1 Methods**

An additional correlational whole-brain analysis was conducted using FreeSurfer’s built-in mri_glmfit function to identify statistically significant effects not captured by the regions of interest-based elastic net analysis. Interindividual differences in local gyrification, in particular, are prone to being averaged out due to localized effects (Schaer *et al.*, 2008, 2012).

For each gray matter feature, general linear models (GLM) were built. In contrast to the ROI analyses, statistical models were estimated at each vertex on the triangle meshes. Per default, cortical features were entered as dependent variables, and a maximum of two covariates could be introduced in the model. In line with past literature, age at the MRI session and gender were added. Two contrasts were tested for each of the three GLMs: First, the association between reading scores and gray matter macrostructure was examined, regressing out sex and age. Second, the difference between the gender literacy slopes regressing out age (i.e., interaction effect) was inspected.

Results were corrected for vertex-wise multiple comparisons using a cluster-based procedure that was adapted for the cortical surface (mri_glmfit-sim; (Ségonne *et al.*, 2004; Hagler, Saygin and Sereno, 2006). This procedure utilizes Monte Carlo simulations of white noise on the cortical surface to obtain a measure of the distribution of the maximum cluster size, given that the null hypothesis is true. A normal distribution z-map was synthesized, applying the same smoothing as the original data. The procedure was repeated over 10.000 iterations. The vertex-wise cluster forming threshold was set to p < 0.01 (- log_10_ (2)). The clusters of the original whole-brain analyses were then assigned a p value representing the probability of finding a maximum cluster of the same size or larger during the permutation simulation.

**3.2 Results**

Overall, this analysis revealed significant associations for gray matter volume and the local gyrification index. None of the gray matter features revealed a significant interaction effect with sex.

Literacy was significantly predicted by the gray matter volume in the right anterior lingual gyrus (p_cluster_ = .044) and cortical folding in the left anterior, dorsal part of the superior parietal gyrus (p_cluster_ < .001). More in-depth information about these clusters can be found in Table 2 and Figure 2. All p values were corrected for multiple comparisons using Monte Carlo simulations as described in section 2.1.

| Gray matter feature | Cluster | Cluster size (mm^2^) | CWP | Peak Talaraich coordinates  X Y Z | Corre-lation |
| --- | --- | --- | --- | --- | --- |
| GMV  LGI | Right lingual gyrus  Left superior parietal gyrus | 966.14  470.27 | .044  .000 | 17.9 -61.2 -2.9  -23.7 -45.4 56.8 | positive  negative |

**Supplementary Table 2.** Clusters in the preliterate brain that significantly predicted literacy ability. Only gray matter volume (GMV) and local gyrification index (lGI) showed a significant association with reading and writing scores. P values were cluster-wise corrected (CWP, p < .01) using Monte Carlo simulations.


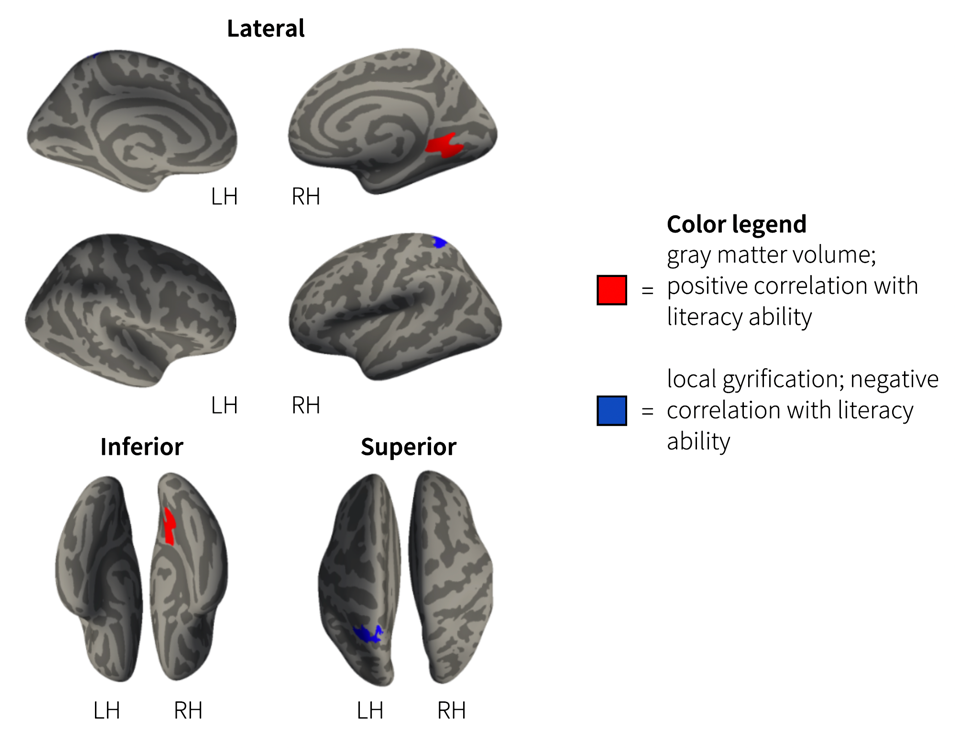


**Supplementary Figure 2.** Clusters in the preliterate brain that significantly predicted literacy ability. The statistical maps are depicted on the left (LH) and right (RH) inflated surface of the FreeSurfer template (fsaverage). Only results that survived a vertex-wise threshold of p < .01, a cluster-wise threshold of p ≤ .05, and correction for multiple comparisons using Monte Carlo simulations are visualized.

**References**

Desikan, R. S. *et al.* (2006) ‘An automated labeling system for subdividing the human cerebral cortex on MRI scans into gyral based regions of interest’, *NeuroImage*. Academic Press, 31(3), pp. 968–980. doi: 10.1016/j.neuroimage.2006.01.021.

Hagler, D. J., Saygin, A. P. and Sereno, M. I. (2006) ‘Smoothing and cluster thresholding for cortical surface-based group analysis of fMRI data’, *NeuroImage*. Academic Press, 33(4), pp. 1093–1103. doi: 10.1016/j.neuroimage.2006.07.036.

Schaer, M. *et al.* (2008) ‘A Surface-based approach to quantify local cortical gyrification’, *IEEE Transactions on Medical Imaging*, 27(2), pp. 161–170. doi: 10.1109/TMI.2007.903576.

Schaer, M. *et al.* (2012) ‘How to measure cortical folding from mr images: A step-by-step tutorial to compute local gyrification index’, *Journal of Visualized Experiments*. Journal of Visualized Experiments, (59), pp. 1–8. doi: 10.3791/3417.

Ségonne, F. *et al.* (2004) ‘A hybrid approach to the skull stripping problem in MRI’, *NeuroImage*. Academic Press, 22(3), pp. 1060–1075. doi: 10.1016/j.neuroimage.2004.03.032.
